# Daniel Webster’s Presence

**Published:** 1882-03

**Authors:** 


### DANIEL WEBSTER'S PRESENCE.

PERHAPS no man ever lived that, quite apart from any adventitious circumstances affecting him, such as accident of birth, or dignity of station, apart indeed from actual achievement of his own, by mere and pure force of inherent character and personality, so impressed the generation to which he belonged as did Daniel Webster. There was something almost supernatural about it. The adjectives by which he was customarily characterized, in the common and instinctive speech of the peopeople, attributed a kind of divinity to the man. He was the "godlike Daniel" to his countrymen in general, who thus called him by a phrase which, with a certain semi-conscious humor in it racy of the national character, redeemed its own excess of veneration by a corrective dash of associated familiarity. But no less the educated men among his fellows were accustomed to employ in their own more scholarly way a similar language. To them, he was "Jove," a "descended god," a "demi-god," "the Olympian." If he went abroad, some Englishman said he "looked like a cathedral," or Sydney Smith, with irreverent homage to his Titan might, said he "was a steam-engine in breeches."

This imposing effect of Webster's personal presence was partly due to the remarkable physical mold in which he was cast. He was not gigantic in proportions, was not even greatly above the medium height; but somehow the beholder took from him an instantaneous and overwhelming impression of immense mass, weight, momentum, in one word, of power. He was always one of the sights of Boston, where his presence in the streets made the neighboring buildings look smaller. Men from the country, that did not know who it was, would stand to gaze at him. Of course, as soon as you were aware that a physical frame so magnificent was the abode of a moral and intellectual nature not unfit to inhabit it, the pleasurable inspiration of wonder and awe that you felt in beholding was more than doubled. But when, in addition, you could further assure yourself that this man was the great lawyer, the great statesman, the great orator of his country and time, why, naturally, the enthusiasm of admiration and delight of which you were conscious in his presence became something extraordinary. CENTURY.

